# 格菲妥单抗治疗复发/难治性B细胞淋巴瘤的疗效及影响因素分析

**DOI:** 10.3760/cma.j.cn121090-20260104-00002

**Published:** 2026-05

**Authors:** 佳 丛, 承稷 王, 娜 姚, 磊 杨, 鑫 李, 进 叶, 晶 杨, 立强 魏, 亮 王

**Affiliations:** 首都医科大学附属北京同仁医院血液内科，北京 100730 Department of Hematology, Beijing Tongren Hospital Affiliated to Capital Medical University, Beijing 100730, China

## Abstract

单中心回顾性分析纳入于北京同仁医院接受格菲妥单抗治疗的32例复发/难治性（R/R）B细胞淋巴瘤患者，以无进展生存（PFS）、总生存（OS）、客观缓解率（ORR）及安全性为研究终点，单因素和多因素Cox回归筛选预后相关因素。中位随访时间为414（95％ *CI*：175～未达到）d，180 d PFS率为39.1％（95％ *CI*：24.0％～63.7％），180 d OS率为72.2％（95％ *CI*：57.5％～90.8％）。格菲妥单抗作为挽救治疗（25例）时，21例可评估患者的ORR为47.6％，完全缓解（CR）率为14.3％。格菲妥单抗用于桥接治疗的6例患者均取得部分缓解。单因素分析结果显示治疗前线数（*HR*＝1.55，95％ *CI*：1.15～2.07，*P*＝0.004）、治疗前接受CAR-T细胞治疗（*HR*＝3.35，95％ *CI*：1.07～10.49，*P*＝0.038）、治疗前接受苯达莫司汀治疗（*HR*＝3.45，95％ *CI*：1.05～11.41，*P*＝0.042）和格菲妥单抗单药治疗（*HR*＝4.18，95％ *CI*：1.42～12.33，*P*＝0.010）是PFS的危险因素。细胞因子释放综合征（CRS）发生率为28.1％，1例患者合并免疫效应细胞相关神经毒性综合征（ICANS），未出现3级以上的CRS和ICANS。格菲妥单抗在R/R B细胞淋巴瘤中展现出了良好潜力，但在多线治疗的患者中单药疗效受限。联合方案更符合临床需求，但需加强不良事件监测和支持治疗。

## Abstract (en)

This single-center retrospective analysis enrolled 32 patients with relapsed/refractory（R/R）B-cell lymphoma who received glofitamab-containing regimens at Beijing Tongren Hospital. Study endpoints were progression-free survival（PFS）, overall survival（OS）, objective response rate（ORR）, and safety. Univariate and multivariate Cox regression analyses were conducted to identify prognostic factors. The median follow-up duration was 414（95％ *CI*: 175–not reached）days, with a 180-day PFS rate of 39.1％（95％ *CI*: 24.0％–63.7％）and a 180-day OS rate of 72.2％（95％ *CI*: 57.5％–90.8％）. The ORR was 47.6％ and the complete response rate（CRR）was 14.3% in 21 evaluable patients among 25 patients who received glofitamab as salvage therapy. All 6 patients who received glofitamab as bridging therapy achieved a partial response. Univariate analysis revealed the number of prior treatment lines（*HR*＝1.55, 95％ *CI*: 1.15–2.07, *P*＝0.004）, prior CAR‑T cell therapy（*HR*＝3.35, 95％ *CI*: 1.07–10.49, *P*＝0.038）, prior treatment with bendamustine（*HR*＝3.45, 95％ *CI*: 1.05–11.41, *P*＝0.042）, and glofitamab monotherapy（*HR*＝4.18, 95％ *CI*: 1.42–12.33, *P*＝0.010）as risk factors for PFS. The incidence of cytokine release syndrome（CRS）was 28.1%, with one patient also developing immune effector cell-associated neurotoxicity syndrome（ICANS）, and no grade of ≥3 CRS or ICANS was observed. Glofitamab demonstrates considerable potential in R/R B-cell lymphoma; however, its efficacy as monotherapy is limited in heavily pretreated patients. Combination regimens may better address clinical needs; however, enhanced monitoring for adverse events and supportive care are warranted.

复发或难治性（R/R）B细胞淋巴瘤的治疗是血液肿瘤领域的重大挑战。对于接受过免疫化疗、挽救化疗甚至嵌合抗原受体T（CAR-T）细胞治疗后仍进展的患者，传统治疗手段的获益极有限[1]。随着治疗线数增加，患者普遍存在功能状态下降和治疗耐受差等问题[2]。CD20×CD3双特异性抗体同时结合肿瘤表面的CD20与T细胞表面的CD3，诱导T细胞肿瘤杀伤，是除CAR-T细胞治疗外的重要免疫治疗选择。格菲妥单抗采用2个CD20结合位点与1个CD3结合位点，在增强肿瘤靶向性和T细胞激活效率方面表现出色[3]–[4]。前瞻性研究显示，在≥2线治疗失败的R/R弥漫大B细胞淋巴瘤（DLBCL）患者中，格菲妥单抗的客观缓解率（ORR）为52％，完全缓解（CR）率为39％，部分患者实现持久缓解[5]–[7]。然而，前瞻性研究入组标准严格，可能无法完全反映真实世界情况，而真实世界回顾性研究患者往往治疗线数更多、疾病负荷更高、合并症更常见[8]–[9]。因此，本研究对我院接受以格菲妥单抗治疗为主的32例R/R B细胞淋巴瘤患者进行单中心回顾性分析，旨在为格菲妥单抗在后线治疗中的真实疗效、安全性及临床管理策略提供补充证据。

## 病例与方法

1. 病例：本研究为单中心、回顾性队列研究，纳入标准：2024年2月至2025年11月期间于北京同仁医院接受以格菲妥单抗为主治疗方案的R/R B细胞淋巴瘤患者（组织病理学确诊），且具有明确病理诊断及可评价的基线、疗效和随访资料。排除标准：合并其他未受控制的恶性肿瘤或严重基础疾病，或关键临床/随访资料缺失、无法进行疗效与生存分析者。研究共纳入32例R/R B细胞淋巴瘤患者，已获北京同仁医院伦理审查委员会批准并豁免患者知情同意（批件号：TREC2022-KY103）。所有涉及人类参与者的研究程序均按照《赫尔辛基宣言》中概述的伦理原则进行。

2. 基线临床信息和评估：所有患者在系统治疗前均接受基线实验室检查和病灶的影像学评估，包括全身PET-CT显像或增强CT检查，中枢神经系统（CNS）受累患者接受颅脑增强MRI检查及脑脊液检查（脑脊液常规、生化和免疫分型等），骨髓受累患者接受骨髓穿刺和病理检查，骨髓标本送检细胞学和免疫分型。疗效按照Lugano 2014标准[10]进行评估；不良事件按不良事件通用术语标准5.0（CTCAE 5.0）评估分级。所有患者在每接受2～4个周期治疗或可疑疾病进展（PD）时接受影像学检查评估治疗后情况，复发和进展的时间点依据影像学与临床评估记录确定；非复发死亡（NRM）计入竞争风险分析中的竞争事件。

3. 治疗方案：所有患者均接受以格菲妥单抗为主的固定疗程方案，最大给药周期为12个周期。第1个周期采用剂量递增策略：2.5 mg，第1天；10 mg，第8天；30 mg，第15天。第2个周期后每21 d静脉注射30 mg，1次。所有患者在格菲妥单抗首剂治疗前7 d接受奥妥珠单抗预处理，剂量为1 000 mg静脉注射。联合方案包括：①GemOx方案（吉西他滨+奥沙利铂）：吉西他滨1 000 mg/m²；奥沙利铂100 mg/m²，均于第1天静脉滴注；21 d为1个周期，根据患者骨髓抑制情况调整剂量。②维泊妥珠单抗：1.8 mg/kg，第1天静脉滴注，21 d为1个周期。③塞利尼索：标准剂量为80 mg口服，每周2次，21 d为1个周期，根据患者胃肠道反应或血细胞减少情况调整至60 mg或40 mg。④匹妥布替尼：200 mg口服，每日1次，根据患者血细胞情况调整剂量。⑤替雷利珠单抗：200 mg静脉滴注，每21 d 1次。⑥维奈克拉：400 mg口服，每日1次，从50 mg起，根据患者血细胞情况调整剂量。⑦米托蒽醌脂质体：18 mg/m²，静脉滴注，21 d为1个周期，根据患者耐受性和骨髓抑制情况调整剂量。

4. 随访及疗效评估：随访截止日期为2025年12月26日。本研究的主要终点为无进展生存（PFS）期，次要终点为总生存（OS）期和ORR，不良事件重点关注格菲妥单抗治疗后细胞因子释放综合征（CRS）、免疫效应细胞相关神经毒性综合征（ICANS）等的发生率和严重程度。PFS期定义为自治疗开始至PD或任何原因死亡的时间；OS期定义为自治疗开始至任何原因死亡的时间。

5. 统计学处理：数据分析和可视化呈现使用R（版本4.5.0）软件进行。计量资料符合正态分布的以*x*±*s*表示，偏态分布的以*M*（范围）表示。计数资料以例数及百分比表示。在单因素Cox分析中，所有*P*值<0.1的变量纳入到多因素Cox比例风险模型构建，针对有限的样本使用coxphf包进行Firth修正。所有检验均为双侧检验，*P*<0.05为差异具有统计学意义。

## 结果

1. 患者基线特征：患者的基线特征见表1。患者总体中位年龄为60.5（38～87）岁，其中≥60岁患者占56.3％（18/32）；男性53.1％（17/32）、女性46.9％（15/32）。累及部位方面，5例患者出现CNS受累，1例患者明确有骨髓累及。胃肠道累及比例为25.0％（8/32），7例患者存在局部骨质侵犯。9例患者存在胸膜或腹膜受累。接受格菲妥单抗治疗的中位周期数为2（1～12）个周期，治疗前的中位治疗线数为4（2～8）。所有的患者基线病理诊断均明确CD20表达阳性，均有抗CD20单抗治疗史。其中3例患者在格菲妥单抗治疗前2个月内再次行病理活检，明确CD20表达阳性；6例患者治疗期间曾接受过DLBCL分子分型检测：N1亚型2例，ST2亚型、A53亚型、MCD亚型和EZB亚型各1例；此外，1例患者检测到MYC断裂重排，无BCL2和BCL6重排。截至末次随访，14例患者因PD停止治疗。

**表1:** 32例复发/难治性B细胞淋巴瘤患者的基线特征［例（％）］

特征	统计值
接受格菲妥单抗治疗时年龄［岁，*M*（范围）］	60.5（38～87）
性别	
男性	17（53.1）
女性	15（46.9）
伴B症状	16（50.0）
肿物直径≥7.5 cm	7（21.9）
Ann Arbor分期	
Ⅰ～Ⅱ期	4（12.5）
Ⅲ～Ⅳ期	28（87.5）
血清LDH水平高于正常上限	20（62.5）
全血淋巴细胞计数低于正常下限	21（65.6）
合并活动性乙肝病毒感染	3（9.4）
IPI评分（分）	
1	3（9.4）
2	5（15.6）
3	8（25.0）
4	12（37.5）
5	4（12.5）
病理类型	
DLBCL，生发中心来源	12（37.5）
DLBCL，非生发中心来源	17（53.1）
高级别B细胞淋巴瘤	1（3.1）
套细胞淋巴瘤	1（3.1）
滤泡性淋巴瘤	1（3.1）
既往治疗	
自体造血干细胞移植	1（3.1）
CD19靶点的CAR-T细胞治疗	15（46.9）
苯达莫司汀	4（12.5）
治疗目的	
挽救治疗	25（78.1）
CAR-T细胞治疗前桥接	6（18.6）
巩固治疗	1（3.1）
治疗方案	
格菲妥单抗单药	12（37.5）
联合GemOx方案	7（21.9）
联合维泊妥珠单抗	4（12.5）
联合塞利尼索	4（12.5）
联合其他化疗或靶向药物	5（15.6）

**注** LDH：乳酸脱氢酶；IPI：国际预后指数；DLBCL：弥漫大B细胞淋巴瘤；CAR-T细胞：嵌合抗原受体T细胞；GemOx：吉西他滨+奥沙利铂

接受格菲妥单抗作为挽救治疗的患者共25例，中位治疗线数为4，其中19例（76.0％，19/25）对一线R-CHOP方案（利妥昔单抗、环磷酰胺、多柔比星、长春新碱和泼尼松）或R-CHOP类方案难治；2例（8.0％，2/25）患者存在CNS受累，14例（56.0％，14/25）患者曾接受过CAR-T细胞治疗。挽救治疗方案选择方面，10例（40.0％，10/25）接受格菲妥单抗单药治疗，6例（24.0％，6/25）联合GemOx方案治疗。基线特征总结见附表1（**扫描本文首页二维码查阅**）。

2. 生存分析和疗效评估：患者总体中位随访时间为414（95％ *CI*：175～未达到）d，中位PFS期为158（95％ *CI*：49～未达到）d，中位OS期为319（95％ *CI*：291～未达到）d；180 d PFS率为39.1％（95％ *CI*：24.0％～63.7％），1年PFS率为32.6％（95％ *CI*：17.8％～59.7％）；180 d OS率为72.2％（95％ *CI*：57.5％～90.8％），1年OS率为31.1％（95％ *CI*：15.5％～62.0％）。以格菲妥单抗作为挽救治疗的25例患者中位PFS期为127（95％ *CI*：43～未达到）d，中位OS期为319（95％ *CI*：259～未达到）d；180 d PFS率为28.7％（95％ *CI*：13.7％～60.2％），1年PFS率为21.6％（95％ *CI*：8.5％～54.7％）；180 d OS率为72.9％（95％ *CI*：56.4％～94.4％），1年OS率为26.7％（95％ *CI*：11.8％～60.7％）。

共28例患者接受了≥1次疗效评估（挽救治疗组21例）。所有患者在90 d、180 d和365 d的累积复发率预估为29.2％、46.8％和53.3％，而挽救治疗组的患者对应时间点的累积复发率为37.6％、62.8％和70.0％。挽救治疗且可评估的21例患者总体ORR为47.6％（10/21），CR率为14.3％（3/21）；其中格菲妥单抗单药治疗的10例患者中仅1例获得部分缓解（PR）且完成8个周期治疗后的再次评估中发生PD。联合GemOx方案治疗的6例患者中，3例因治疗周期短未进行疗效评估；1例患者NRM；余2例已评估的患者，1例达CR、1例达PR，截至末次随访均未见PD。

合并CNS受累的患者共有5例，其中3例选择格菲妥单抗作为CAR-T细胞治疗前的桥接治疗，均取得PR；2例作为挽救治疗，其中1例广泛累及，疾病迅速进展于13 d后死亡，而另1例患者取得PR，随访91 d仍处于缓解状态。6例桥接治疗的患者均取得PR。其中1例患者在挽救化疗达到CR后接受6个周期格菲妥单抗作为巩固治疗，截至末次随访，缓解状态已维持637 d。

3. 危险因素评估：单因素Cox分析显示，治疗前线数（*HR*＝1.55，95％ *CI*：1.15～2.07，*P*＝0.004）、治疗前接受CAR-T细胞治疗（*HR*＝3.35，95％ *CI*：1.07～10.49，*P*＝0.038）、治疗前接受苯达莫司汀治疗（*HR*＝3.45，95％ *CI*：1.05～11.41，*P*＝0.042）和格菲妥单抗单药治疗（*HR*＝4.18，95％ *CI*：1.42～12.33，*P*＝0.010）是PFS的危险因素；治疗前线数也是OS的危险因素（*HR*＝1.49，95％ *CI*：1.06～2.11，*P*＝0.023）。多因素Cox分析显示，格菲妥单抗单药治疗是PFS的独立不良影响因素（*HR*＝4.09，95％ *CI*：4.04～1×10^6^，*P*＝0.010），未发现与OS相关的独立影响因素（*P*值均>0.05）。具体分析结果详见附表2（**扫描本文首页二维码查阅**）。

4. 不良事件：32例患者中9例（28.1％）发生1～2级的CRS、1例发生ICANS，未观察到出现3～4级的CRS或ICANS，无患者因CRS而使用托珠单抗；11例使用地塞米松治疗的患者中有3例因CRS或可疑CRS而使用激素，1例因ICANS使用激素治疗，其余为淋巴瘤治疗用药。治疗期间有4例患者发生NRM，其中1例患者死于继发急性髓系白血病，2例患者死于重症感染，1例患者死于上消化道出血。血液学不良反应是最常见的不良事件（78.1％，25/32）。基线血象方面，13例患者存在中性粒细胞减少，11例患者存在血小板减少，其中7例患者同时存在以上两者，但所有患者在治疗前均未达到中性粒细胞缺乏。9例患者发生肺部感染，其中2例患者为重症肺炎，因感染进展死亡，1例为多重耐药菌感染，1例为疱疹病毒感染。胃肠道系统不良事件中，1级腹泻、纳差和3级腹泻、纳差各有1例。循环系统的2例不良事件均为一过性心房颤动。

## 讨论

本队列研究显示格菲妥单抗作为挽救治疗，尽管部分联合其他治疗，疗效仍与前瞻性试验有差距，21例可评估的挽救治疗患者，ORR为47.6％，CR率为14.3％；单药治疗时仅1例患者取得PR。多项真实世界研究显示ORR为34％～54％，CR率为14％～30％[8],[10]–[15]。这并非否认格菲妥单抗的疗效，而是临床上面临多线治疗的患者更多[8]，其肿瘤负荷更大、功能状态更差[14]，如NP30179入组要求ECOG评分为0～1分，而本队列仅有12例（48.0％）挽救治疗的患者达到该要求。体能状态差提示患者对CRS、ICANS等不良反应调节能力弱，免疫微环境也存在衰退[16]–[17]。REALBiTe研究中71.0％的患者不符合注册临床试验的准入标准，而研究指出试验不符合性是导致生存期缩短的独立预测因素[13]。

除多线治疗外，我们发现治疗前接受CAR-T细胞治疗和接受苯达莫司汀治疗是预后的危险因素。Brooks队列发现，CAR-T细胞治疗失败后接受格菲妥单抗或艾可瑞妥单抗（epcoritamab）的患者的中位PFS期仅2.5个月[18]。原因可能是经过大规模扩增和回输后的炎症风暴，T细胞耗竭并形成免疫抑制的微环境，从而阻断格菲妥单抗对T细胞的再激活[19]。LYSA研究表明，在CAR-T细胞治疗失败后接受格菲妥单抗治疗，ORR为76.1％，中位PFS期仅3.8个月，治疗结束时只有13例（28.3％）患者仍维持缓解[20]。苯达莫司汀是后线治疗的常用药物，对免疫功能尤其是CD4^+^T细胞具有持久的抑制作用[21]。Shumilov等[11]研究显示格菲妥单抗治疗前6个月内接受过苯达莫司汀治疗的患者预后更差。以上提示免疫微环境是格菲妥单抗发挥作用的重要因素，免疫治疗应更早应用于R/R患者。STARGLO研究发现，相较于R-GemOx方案，格菲妥单抗联合GemOx方案作为挽救化疗表现出更长的生存期（中位PFS期：13.8个月对3.6个月；中位OS期：25.5个月对12.9个月）[22]；安全性也可耐受[23]。另一项回顾性分析显示，格菲妥单抗联合GemOx方案的ORR为78％（21/27），CR率为41％（11/27）；其中7例患者接受该方案作为CAR-T细胞治疗前的桥接方案，ORR为85.7％[24]。Brody等[25]报道艾可瑞妥单抗联合GemOx方案治疗103例R/R B细胞淋巴瘤，ORR为85％，CR率为61％；3级以上CRS发生率仅为1％。

格菲妥单抗联合维泊妥珠单抗的ORR为78.3％，CR率为59.7％，中位PFS期和OS期分别为12.3和33.8个月[26]。Diefenbach等[27]报道格菲妥单抗联合R-ICE方案（利妥昔单抗+异环磷酰胺+卡铂+依托泊苷）治疗R/R DLBCL，32例可评估疗效的患者CR率为68.7％。格菲妥单抗联合来那度胺及布鲁顿酪氨酸激酶抑制剂治疗，80例可评估的患者ORR为82.5％，CR率为53.8％；6个月PFS率为62.8％[28]。国内一项试点试验中，10例接受格菲妥单抗联合替雷利珠单抗的患者，其中5例取得CR、1例PR，5例患者的缓解时间超过12个月[29]。本研究也提示联合治疗方案缓解率更高。联合GemOx方案或维泊妥珠单抗是相对成熟的方案，而其他化疗方案或靶向药物有待于进一步的探索。

不良反应方面，血液学不良事件最常见。多线治疗后骨髓造血功能受损，28％的患者在治疗前已经同时存在中性粒细胞和血小板减少；且格菲妥单抗和化疗均会增加血液学不良反应的发生[5],[22]，尤其是已存在血细胞减少的患者。本队列中CRS和ICANS发病率相对低，可能与糖皮质激素的使用相关[30]，而激素的使用是为了协助控制病情而非预防CRS。

综上，在R/R B细胞淋巴瘤患者中格菲妥单抗联合方案较单药呈现出更优的疾病控制趋势。本研究补充了格菲妥单抗在中国真实世界多线治疗人群中的应用证据，以及其在联合治疗策略中的优势，同时也需谨慎地评估疗效和监测不良事件。本研究为单中心研究，例数较少，疗程短，未来仍需多中心、大样本量的研究进一步验证。

## Supplementary Material


