# Supplementary figures and images for: Glofitamab for relapsed/refractory B-cell lymphoma: analysis of efficacy and influencing factors
Source: Zhonghua Xue Ye Xue Za Zhi. 2026 May;47(5):480–4. [Article in Chinese] doi: 10.3760/cma.j.cn121090-20260104-00002 (PMC13416538; doi:10.3760/cma.j.cn121090-20260104-00002)

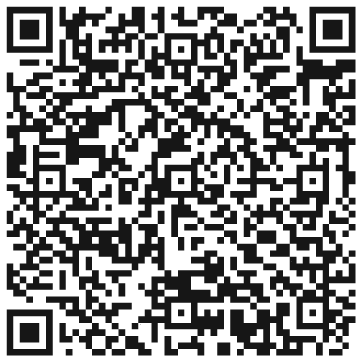

Supplement: Supplementary file 1 [file cjh-47-05-480-g001.tif]
